# Colonization of the lower urogenital tract with *Ureaplasma parvum* can cause asymptomatic infection of the upper reproductive system in women: a preliminary study

**DOI:** 10.1007/s00404-013-3102-7

**Published:** 2013-12-07

**Authors:** Kasprzykowska Urszula, Elias Joanna, Elias Marek, Mączyńska Beata, Sobieszczańska Beata Magdalena

**Affiliations:** 1 Department of Microbiology, University of Medicine, Chałubińskiego 4 Street, 50-346 Wrocław, Poland; 2 Gynecology Clinic, 2nd Department of Gynecology and Obstetrics, Wrocław University of Medicine, Wrocław, Poland

**Keywords:** *Ureaplasma parvum*, *Ureaplasma urealyticum*, Upper urogenital tract, Asymptomatic infection

## Abstract

**Purpose:**

Genital ureaplasmas are considered opportunistic pathogens of human genitourinary tract involved in adverse pregnancy sequelae and infertility. While association of *Ureaplasma urealyticum* with urogenital tract infections is well established, the role of *Ureaplasma parvum* in these infections is still insufficient. In the study, we compared how often cervicovaginal colonization with *U. parvum* is associated with the presence of these microorganisms in the upper genitourinary tract of fertile and infertile women.

**Methods:**

We used PCR assay to determine the prevalence of *U. parvum* and *U. urealyticum* in pairs of specimens, i.e., vaginal swabs and Douglas’ pouch fluid samples from consecutive 40 women with no symptoms of genital tract infection.

**Results:**

In total, 19 (47.5 %) of the 40 samples were positive for ureaplasmas. *U. parvum* was simultaneously detected in pairs of samples in five (55.5 %) of the nine (47.4 %) women positive in PCR assay. As many as 5 (18.5 %) of the 27 infertile women and 1 (7.7 %) of the 13 fertile women showed infection of the upper genital tract with *U. parvum*.

**Conclusion:**

The results of the study demonstrated that colonization of the lower genital tract with *U. parvum* can produce asymptomatic infection of the upper reproductive system in women. These findings also imply that *U. parvum* may be present in the upper genital tract at the time of conception and might be involved in adverse pregnancy outcomes.

## Introduction

*Mycoplasmataceae* family comprises pleomorphic, cell wall deficient bacteria of *Mycoplasma* and *Ureaplasma* genera, referred to collectively as mycoplasmas [1, 2]. Genital ureaplasmas, i.e., *U. urealyticum* (biovar 2 comprising serotypes 2, 4, 5, 7 and from 7 to 13) and *U. parvum* (biovar 1, serotypes 1, 3, 6 and 14) are considered natural inhabitants of the lower urogenital tract of humans as they are often isolated from healthy individuals [3, 4]. On the other hand, ureaplasmas are involved in a variety of infections in humans. There are several reports confirming the association of *U. urealyticum* with nongonococcal urethritis, infertility, postpartum endometriosis, chorioamnionitis, spontaneous abortion, stillbirth, premature birth and perinatal morbidity and mortality [1, 2, 5]. While association of *U. urealyticum* with urogenital tract infections is well established, the role of *U. parvum* in these infections is still insufficient and underestimated, mostly because of lack of ureaplasma species differentiation. Nevertheless, the isolation of *U. parvum* from subjects with genitourinary tract infections as well as findings of studies on laboratory animals seems to confirm the pathogenicity of the species [6]. De Francesco et al. [7] have found *U. parvum* serovar 3/14 in 86 % of women with symptomatic genital tract infections. Similarly, Kong et al. [8] have identified *U. parvum* in vaginal swabs of 87 % pregnant women. *U. parvum* has also been linked with adverse pregnancy outcomes such as late abortion and early preterm birth [9]. Kacerovsky et al. [10] identified *U. parvum* in 57 % of healthy nonpregnant women and the organism was far more prevalent than any of the other genital mycoplasmas, *Chlamydia* spp. or viruses.

The pathomechanism of infections caused by genital ureaplasmas is not fully elucidated;however, several virulence factors, i.e., IgA protease, phospholipases A and C as well as hemolytic activity and attachment to the host cell have been described in *Ureaplasma* species. Moreover, it has been showed that long-lasting colonization of mucosal membranes of the lower urogenital tract and immune response to ureaplasmal antigens stimulated inflammation demonstrated by elevated levels of proinflammatory cytokines e.g., interleukin-6 (IL-6) and IL-8 [5, 11].

Most studies linking ureaplasmas with genital tract infections, adverse pregnancy outcomes or infertility are limited to sampling the lower genital tract, thus yielding inconclusive results and complicating an understanding the potential role of *U. parvum* in the reproductive tract infections. The study was undertaken to elucidate whether the colonization of the lower urogenital tract with *U. parvum* can produce ascending asymptomatic infection of the upper genital tract in nonpregnant fertile and infertile women. In the study, PCR assay was used to determine the prevalence of *U. parvum* and *U. urealyticum* in pairs of specimens, i.e., vaginal swabs and Douglas’ pouch fluid samples obtained from consecutive 40 women with no symptoms of infection of the urogenital tract that were subjected to diagnostic laparoscopy.

## Materials and methods

### Patients’ specimens

The study was carried out on pairs of specimens obtained from a consecutive 40 women (aged 22–38 years) living in Lower Silesia region, Poland, that were admitted to the Gynecology Clinic at the Department of Gynecology and Obstetrics of Wrocław University of Medicine for diagnostics laparoscopy. A detailed gynecological examination and a general physical examination were performed on all women, which made it possible to exclude women with symptoms of reproductive system infection. The primary infertility was diagnosed in 27 women among whose 14 (51.8 %) had unexplained or idiopathic infertility, six (22.2 %) suffered from tubal obstruction diagnosed by laparoscopy, three (11.1 %) presented ovulation disorders and endometriosis, and four (14.8 %) had no overt abnormalities but whose partners showed sperm irregularities that were suspected of preventing conception. Thirteen women with proven fertility (history of giving birth to at least one child) had diagnosed laparoscopically benign ovarian tumors and uterine myomas. Cervical swabs were collected using a sterile flocked swabs (Copan, Italy) and transferred into one-tube DNA purification system (OTP—Reagent, Bioron, Germany) container. A 1 ml of fluid aspirated from the pouch of Douglas was transferred into sterile tube and immediately frozen at −20 °C. Samples were transported to the laboratory within 1–3 h after collection. In both specimens obtained from every woman the presence of common urogenital pathogens e.g., *Chlamydia trachomatis*, *Neisseria gonorrhoeae*, group B streptococci, was excluded by specific microbiological examination.

### PCR assay

Genomic DNA from cervical swabs was extracted using commercial kit according to OTP—Reagent protocol. The resultant DNA was frozen at −20 °C for further analysis. DNA isolation from frozen Douglas’ pouch fluid was carried out using QIAamp DNA kit (Qiagen, Hilden, Germany) in accordance with supplier’s protocol. GeneProof Mycoplasma species PCR IVD CE kit (GeneProof, Czech Republic) was used to detect all clinically significant human mycoplasmas and ureaplasmas. In brief, PCR was performed in 40 μl volumes containing 4 μl of isolated DNA sample and 36 μl of an optimized ready-to-use solution of MasterMix at the following conditions: UNG was activated for 2 min at 37 °C followed by 15 min at 96 °C of DNA denaturation. PCR amplification consisted of 45 cycles at 95 °C for 1 min; 69 °C for 15 s; and 68 °C for 30 s. Final extension step was carried at 72 °C for 5 min. Samples positive for *Mycoplasma* spp. were further investigated for ureaplasmas using GenePack DNA PCR tests: *U. parvum* biovar 1 (Upa) and *U. urealyticum* biovar 2 (Uur) (Bioron, Germany). All reagents required for this PCR (Taq DNA polymerase, dNTPs, specific primers, salts, and stabilizers) were lyophilized in PCR tubes by manufacturer, for final reaction volume of 20 μl. 3 μl of DNA sample, 10 μl specific buffer provided in the kit, and 7 μl of PCR grade sterile water, were added. Amplification was performed under the following conditions: 95 °C for 2 min, followed by 45 cycles of 95 °C for 1 min, 58 °C for 40 s and 74 °C for 1 min. A final elongation step was performed at 74 °C for 2 min. All PCR amplifications were performed in a DNA—Engine PT200 thermal cycler (MJ Research Waltham, MA, USA). PCR products were visualized after electrophoresis on a 2 % agarose gel in Tris-acetate–EDTA buffer by staining with ethidium bromide.

## Results

Altogether, 21 (52.5 %) of the 40 samples were positive with *Mycoplasma* spp. and 19 (47.5 %) samples were negative. Among these 21 positive for genital *Mycoplasma* specimens, as many as 19 (90.5 %) showed positive PCR reaction for ureaplasmas: nine (42.8 %) for *U. parvum* and ten (47.6 %) for *U. urealyticum.* There was no samples positive for these both *U. parvum* and *U. urealyticum* species. The remaining two (9.5 %) samples represented other than ureaplasmas genital *Mycoplasma* species.

Colonization of the lower genitourinary tract with *U. parvum* was shown eight, (42.1 %) women among whom six (66.7 %) had also positive PCR result in Douglas’ pouch fluid samples. *U. urealyticum* was simultaneously detected in pairs of samples in seven (70 %) out of the ten (52.6 %) women infected with the microorganism (Tables 1, 2, 3). These results indicated that in more than 60 % of women carrying of *U. parvum* or *U. urealyticum* in the lower genitourinary tract ascending infection of the upper, sterile parts of the genitourinary tract occurred.

**Table 1:** The occurrence of positive and negative PCR reactions among samples from infertile and fertile women

Sample origin	*Mycoplasma* spp.	*U. parvum*	*U. urealyticum*
Infertile women (*n* = 27)			
Idiopathic infertility (*n* = 14)			
1	+	–	+
2	–	–	–
3	–	–	–
4	+	+	–
5	+	+	–
6	+	–	–
7	+	–	+
8	+	+	–
9	–	–	–
10	+	+	–
11	+	–	+
12	–	–	–
13	+	–	–
14	–	–	–
Tubal obstruction (*n* = 6)			
1	+	–	+
2	+	–	–
3	–	–	–
4	–	–	–
5	–	–	–
6	+	–	+
Ovulation disorders, endometriosis (*n* = 3)			
1	+	+	–
2	+	+	–
3	–	–	–
Sperm irregularity (*n* = 4)			
1	–	–	–
2	–	–	–
3	+	+	–
4	+	–	+
Fertile women (*n* = 13)			
1	+	+	–
2	+	–	+
3	–	–	–
4	–	–	–
5	–	–	–
6	–	–	–
7	–	–	–
8	–	–	–
9	+	–	+
10	–	–	–
11	+	+	–
12	+	–	+
13	–	–	–

**Table 2:** The prevalence of *U. parvum* and *U. urealyticum* in 19 ureaplasma-positive paired samples

No. of positive samples	*U. parvum*	*U. urealyticum*	Total
Total	9 (47.4 %)	10 (52.6 %)	19 (100 %)
Cs	8 (42.1 %)	10 (52.6 %)	18 (94.7 %)
Df	6 (31.6 %)	7 (36.8 %)	13 (68.4 %)
Cs + Df	5 (26.3 %)	7 (36.8 %)	12 (63.1 %)

*Cs* cervical swab,* Df* Douglas pouch fluid

**Table 3:** The prevalence of *U. parvum* and *U. urealyticum* in paired samples from fertile and infertile women

Ureaplasma species	Infertile women *n* = 27 (67.5 %)	Fertile women *n* = 13 (32.5 %)	Total *n* = 40 (100 %)
*U. parvum*			
Total	7 (25.9 %)	2 (15.4 %)	9 (22.5 %)
Cs	6 (22.2 %)	2 (15.4 %)	8 (20 %)
Df	5 (18.5 %)	1 (7.7 %)	6 (15 %)
Cs + Df	4 (14.8 %)	1 (7.7 %)	5 (12.5 %)
*U. urealyticum*			
Total	7 (25.9 %)	3 (23.1 %)	10 (25 %)
Cs	7 (25.9 %)	3 (23.1 %)	10 (25 %)
Df	5 (18.5 %)	2 (15.4 %)	7 (17.5 %)
Cs + Df	5 (18.5 %)	2 (15.4 %)	7 (17.5 %)

*Cs* cervical swab,* Df* Douglas pouch fluid

In the study, we also analyzed if there is any relationship between the infection of the upper genitourinary tract caused by ureaplasmas and infertility (Table 3). Infection of the upper genitourinary tract was confirmed by the detection of *U. parvum* in five (18.5 %) of the 27 infertile women and in one (7.7 %) of the 13 fertile women. Similar result was related to *U. urealyticum*. Although, there was no statistical differences between these two groups of women, infection of the upper genitourinary tract was more common among infertile women (*p* > 0.05).

Generally, the results of the study showed that *U. parvum* can produce asymptomatic infections of the upper genital tract in women carrying these organisms in the lower urogenital tract as frequently as *U. urealyticum*.

## Discussion

Albeit the implication of ureaplasmas in lower urogenital tract infections and their adverse effect on pregnancy seem to be well established, their role in the upper genitourinary tract infection is not so obvious. Difficulty in the routine detection of ureaplasmas caused by their fastidious growth requirements, but also lack of susceptible and rapid assays as well as need to obtain specimens from the upper genitourinary tract using invasive procedures, make the relationship of ureaplasmas with these infections underestimated [1, 2]. There are many reports linking ureaplasmas with infertility. Indeed, genital ureaplasmas are isolated more commonly from infertile subjects than than from healthy controls [5, 12, 13].

The association of genital ureaplasmas with a variety of pathological conditions in women is based on the detection of these potential pathogens in the specimens from the lower genitourinary tract e.g., vaginal or endocervical swabs. However, there is little if any evidence documenting the detection of ureaplasmas in the sterile samples from the upper genitourinary tract of asymptomatic women. Colonization of the vagina or cervix with ureaplasmas can lead to the ascending infection of the upper genital tract with subsequent inflammation of these structures. Long-lasting inflammation within upper genital tract can cause scarring of the inflamed tissue and can contribute to the development of reproductive system disorders such as infertility [5].

While the association of *U. urealyticum* with the urogenital tract infections is well established, the role of *U. parvum* in these infections is still insufficient. Moreover, the presence of *U. parvum* in the lower urogenital tract of many healthy nonpregnant women complicates an understanding of the potential role of these microorganisms in adverse outcomes of pregnancy and reproductive system disorders. In the study we have compared the prevalence of *U. parvum* and *U. urealyticum* in pairs of specimens, i.e., vaginal swabs and Douglas’ pouch fluid samples obtained during the laparoscopy from consecutive 40 women with no symptoms of the urogenital tract infection. Both these species, *U. parvum* and *U. urealyticum* were detected in Douglas’ pouch fluid samples in about 60 % of women carrying ureaplasmas in the lower urogenital tract, confirming that cervicovaginal colonization can lead to the direct ascent of ureaplasmas to the sterile upper reproductive tract. To our knowledge this is the first report on the detection of *U. parvum* in Douglas’ pouch fluid samples from women without any signs of the upper genital tract infection. The findings of the study indicated that *U. parvum*, similarly to *U. urealyticum*, can produce asymptomatic infection of the upper genital tract in women. The presence of these organisms in the sterile part of the genital tract may induce long-lasting subacute inflammation that may be the cause of the upper genital tract disorders e.g., infertility. Zhu et al. [14] in the study of pathogenicity of *U. parvum* serotypes 1, 3, and 6, and *U. urealyticum* serotypes 4 and 8 for BALB/c female mice showed that all these serotypes caused morphological changes of the external genitalia associated with infiltration by inflammatory cells and increased tumor necrosis factor-α (TNF-α) expression. Similarly, Shimizu et al. [15] indicated that *U. parvum* lipoproteins activated nuclear factor NF-κB thus inducing the synthesis of tumor necrosis factor alpha (TNF-α) in mouse peritoneal macrophages. Allam et al. [6] in their study on bladder tissue from animals actively colonized by *U. parvum* demonstrated significant alterations in actin binding proteins that may influence cell signaling cascades regulating cell motility, differentiation, apoptosis, and inflammation. Moreover, they have shown in a rats experimentally infected with *U. parvum* three clinical outcomes of infection, i.e., clearance of infection within 2 weeks in one-third of animals, then asymptomatic infection and acute, complicated infection of bladder associated with the presence of ureaplasmas within bladder submucosa. Their study pointed out that the effect of infections caused by *U. parvum* depends on the host-associated factors. Most localized to the lower urogenital tract infections caused by ureaplasmas characterize asymptomatic or subclinical course due to limited immune response. On the other hand, there are several reports on the disseminated, acute ureaplasmal infections that occur mostly among neonates and immunosuppressed individuals, indicating the significance of the host immune response in the clinical outcome of these infections [16–19]. All these reports confirmed pathogenicity of *U. parvum* for the host cells and indicated that the occurrence of these organisms in the genitourinary tract is associated with inflammatory response. Demonstrated in the study, the presence of *U. parvum* and *U. urealyticum* in the Douglas’ pouch, even asymptomatic, implies that immune response against these organisms seem to be inevitable and may produce chronic inflammation with subsequent development of abnormalities within upper genitourinary tract of women.

In addition, in the study we compared the prevalence of both, *U. parvum* and *U. urealyticum* in the specimens obtained from fertile and infertile women. The results showed that the upper genitourinary tract infection with *Ureaplasma* spp. was more common among infertile than fertile women what is in concordance with Dhawan et al. [13] who have shown the predominance of *U. parvum* serovar 3/14 in patients with genital infections and infertility.

The results of the study also raised the problem of treating ureaplasma-positive individuals. Taking into consideration pathogenic potential of these bacteria, and the fact that most infections caused by *Ureaplasma* spp. are mild or unnoticeable, and that the colonization of the lower genitourinary tract with ureaplasmas is common, general rules should be established.

Since many investigators have shown using quantitative techniques that the degree of colonization with *Ureaplasma* spp. is correlated with clinical presentation and adverse outcomes [4, 20], the decision whether the presence of ureaplasmas in the genitourinary tract should be treated depends on the specimen and the concentration of ureaplasmas in the specimen. In case of specimens obtained from the lower genitourinary tract colonized by normal flora, culture or/and commercially available diagnostic kits should be used instead of sensitive molecular methods. Many available screening tests allow to detect ureaplasmas and, what is more important, to establish their load. The concentration of ureaplasmas in the specimen from the lower genitourinary tract amounting ≥10^4^ organisms per ml is commonly accepted as the load indicating an infection that should be treated with antimicrobials. Lower concentrations (<10^4^ organisms per ml) of ureaplasmas in all specimens from the lower genitourinary tract are considered colonization that does not need treatment. In case of specimens obtained from the upper genitourinary tract, the load of ureaplasmas is not as important as in case of specimens from the lower genitourinary tract as the occurrence of ureaplasmas in the sterile body sites is always considered an infection that should be treated. Thus, the detection of ureaplasmas in all specimens obtained from the upper genitourinary tract should rely on sensitive molecular methods that allow detecting even low concentrations of ureaplasmas within specimens as well as enabling their identification to the species level. Moreover, treatment should include infection in both, men and women and their sexual contacts.
